# Mechanisms and Clinical Trials of Hepatocellular Carcinoma Immunotherapy

**DOI:** 10.3389/fgene.2021.691391

**Published:** 2021-07-08

**Authors:** Shao-Li Huang, Yu-Ming Wang, Quan-Yue Wang, Guang-Gui Feng, Fu-Qing Wu, Liu-Ming Yang, Xi-He Zhang, Hong-Wu Xin

**Affiliations:** ^1^Department of Clinical Laboratory, Lianjiang People’s Hospital, Zhanjiang, China; ^2^Doctoral Scientific Research Center, Lianjiang People’s Hospital, Zhanjiang, China; ^3^Guangdong Medical University Affiliated Lianjiang People’s Hospital, Zhanjiang, China; ^4^Department of Spinal and Neural Functional Reconstruction, Beijing Bo’ai Hospital, China Rehabilitation Research Center, Beijing, China; ^5^School of Rehabilitation Medicine, Capital Medical University, Beijing, China; ^6^Qinghai Institute of Health Sciences, Xining, China; ^7^Department of Gastroenterology and Hepatology, Lianjiang People’s Hospital, Zhanjiang, China; ^8^Laboratory of Oncology, Center for Molecular Medicine, School of Basic Medicine, Faculty of Medicine, Yangtze University, Jingzhou, China; ^9^Department of Biochemistry and Molecular Biology, School of Basic Medicine, Faculty of Medicine, Yangtze University, Jingzhou, China

**Keywords:** hepatocellular carcinoma, immunotherapy, bispecific T-cell Engagers, bispecific antibody, aptamer, vascular endothelial growth factor (VEGF)

## Abstract

Hepatocellular carcinoma (HCC), one of the most common and lethal tumors worldwide, is usually not diagnosed until the disease is advanced, which results in ineffective intervention and unfavorable prognosis. Small molecule targeted drugs of HCC, such as sorafenib, provided only about 2.8 months of survival benefit, partially due to cancer stem cell resistance. There is an urgent need for the development of new treatment strategies for HCC. Tumor immunotherapies, including immune check point inhibitors, chimeric antigen receptor T cells (CAR-T) and bispecific antibodies (BsAb), have shown significant potential. It is known that the expression level of glypican-3 (GPC3) was significantly increased in HCC compared with normal liver tissues. A bispecific antibody (GPC3-S-Fabs) was reported to recruit NK cells to target GPC3 positive cancer cells. Besides, bispecific T-cell Engagers (BiTE), including GPC3/CD3, an aptamer TLS11a/CD3 and EpCAM/CD3, were recently reported to efficiently eliminate HCC cells. It is known that immune checkpoint proteins programmed death-1 (PD-1) binding by programmed cell death-ligand 1 (PD-L1) activates immune checkpoints of T cells. Anti-PD-1 antibody was reported to suppress HCC progression. Furthermore, GPC3-based HCC immunotherapy has been shown to be a curative approach to prolong the survival time of patients with HCC in clinically trials. Besides, the vascular endothelial growth factor (VEGF) inhibitor may inhibit the migration, invasion and angiogenesis of HCC. Here we review the cutting-edge progresses on mechanisms and clinical trials of HCC immunotherapy, which may have significant implication in our understanding of HCC and its immunotherapy.

## Introduction

Hepatocellular carcinoma (HCC) is caused by genetic and epigenetic changes of tissue stem cells and progressed through the interaction between cancer cells and tumor microenvironment (Hari et al., 2011; Xin et al., 2012, 2016; Xin et al., 2013; Xin H. W. et al., 2013; Ellison et al., 2017; Liu et al., 2017). Infection with hepatitis B virus and hepatitis C virus is the major cause of chronic hepatitis and HCC. Besides, aflatoxin, smoking, obesity, excessive alcoholic drink and hyperlipidemia also play significant roles in the process of HCC. Consequently, high incidence and mortality rate have made HCC to be one of the deadliest cancers and severe health issue (Guo et al., 2018; Hu et al., 2018; Jin et al., 2018).

Hepatocellular carcinoma progresses with no clinical symptoms in the early stage, whereas clinical symptoms become obvious in the advanced stage, leading to ineffective intervention and poor prognosis. Current prevailing medical treatments for HCC, including surgical ablation, chemotherapy (including chemoembolization), radiotherapy (including proton beam therapy), targeted therapy, and virotherapy, can generally achieve limited overall survival time (Xiang et al., 2015). The effective approach for the treatment of HCC is excision of cancerous tissue in early phases. The target selectivity of radiotherapy for HCC patients is not sensitive enough (Zhu et al., 1998). Sorafenib, an multi-kinase inhibitor, is one of the recommended medicine for patients with advanced HCC and has been shown to improve the overall survival, but with various side effects such as diarrhea, fatigue, and skin reaction of hand and foot. Furthermore, the drug resistance is the major issue for the treatment of HCC patients at advanced stages. Only 30% of HCC patients obtain survival benefits from sorafenib (Su et al., 2018). Other multikinase inhibitors such as lenvatinib and regorafenib have also been approved for the treatment of HCC patients. Lenvatinib is approved as the first-line therapy and regorafenib, which is an inhibitor closely associated with sorafenib, is approved as the second-line therapy. However, lenvatinib and regorafenib have limited survival benefit for the patients with HCC (Ruiz De Galarreta et al., 2019). In addition, cancer stem cells (CSCs) display cellular hierarchies with self-renewing tumor-initiating cells at the apex and are believed to cause drug resistance of tumors (Yamashita and Wang, 2013; De Angelis et al., 2019). Studies have demonstrated that ten of label-retaining cancer cells (LRCC) of human HCC are able to initiate tumors. Unfortunately, LRCC is relatively resistant to sorafenib and metformin (a reported potential drug against CSC) (Xin H. W. et al., 2013).

**FIGURE 1:**
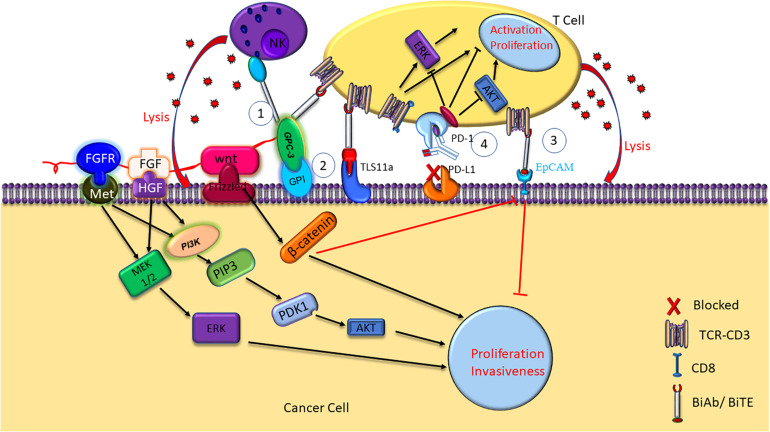
Mechanisms of HCC immunotherapy targeting GPC3, TLS11a, EpCAM, and PD-1. NK BsAb and BiTE mediate T cell immunotherapy targeting GPC3 + HCC. GPC3 promotes HCC growth by upregulating the Wnt-β-catenin and FGF signaling. Aptamer bispecific antibody TLS11a/CD3 can mediate T cell immunotherapy by binding to both the HCC cancer cell and the T cell, inducing T cell cytotoxicity and proinflammatory cytokine release, such as IL-2, IL-6, IL-10, TNF-α, and IFN-γ. BiTE mediates T cell immunotherapy targeting EpCAM + HCC. Antibodies against PD-1 or PD-L1/2 on the cell surface inhibit the immune check point, and block the immune escape in HCC.

Therefore, there is an urgent need for effective therapeutic strategies for HCC. Recently, tumor immunotherapies, including immune check point inhibitors, chimeric antigen receptor T cells (CAR-Ts), and bispecific antibodies (BsAb), have shown great clinical benefit for HCC patients. BiTE, a form of BsAb that binds CD3 and tumor-associated antigens (TAA) (Frankel and Baeuerle, 2013; Sedykh et al., 2018), capable to recruit T cells to cancer cells for elimination (Oberst et al., 2014). The specific killing of the cancer cells by BiTE was mediated by concomitant cytokine release and HCC cell lysis (Figure 1). Here we review recent research progresses in the mechanisms and clinical trials of HCC immunotherapies against glypican-3 (GPC3), epithelial cell adhesion molecule (EpCAM) and TLS11a, and programmed death-1 (PD-1).

## Mechanisms of HCC Immunotherapy

### GPC3-Based Immunotherapy: GPC3/NK BsAb, GPC3/BiTE, GPC3/CAR-Ts and GPC3 Peptide Vaccine

Glypican-3 belongs to heparin sulfate (HS) protein polysaccharide family and anchors to cell surface by glycosylphosphatidylinositol (GPI) (Filmus and Selleck, 2001; Sun et al., 2017). Glypicans interact with growth factors and play significant roles in cell proliferation, differentiation, and migration (Filmus and Selleck, 2001). The growth of HCC cells can be stimulated by GPC3 by the means of typical Wnt signaling pathway (Figure 1; Capurro et al., 2005; Chen et al., 2018). A frizzled-like cysteine-rich domain of GPC3 can regulate Wnt binding and mediate the growth of mouse HCC tumors (Li et al., 2019). Besides, GPC3 can negatively regulate bone morphogenesis protein 7 (BMP-7) to modulate cell proliferation (Midorikawa et al., 2003; Chen et al., 2018). Overexpression of GPC3 in the cell membrane can induce M2-polarized tumor-associated macrophages to enter human HCC tissues that may stimulate the progression and metastasis of HCC (Takai et al., 2009). GPC3 is absent in normal and benign tissues, whereas expresses in human embryo and many kinds of neoplastic cells such as HCC, melanoma, and squamous non-small cell lung cancer (Sun et al., 2017). GPC3 accounts for the initiation and progression of HCC (Llovet et al., 2006; Baumhoer et al., 2008). A meta-analysis found that GPC3 was highly expressed in high-grade and advanced stage HCC cells, and tumor vascular cells in HCC (Liu H. et al., 2018). The expression of GPC3 is relate with tumor size of HCC, which suggest that GPC3 may potentially become an early diagnosis biomarker of HCC. Research has suggested that the accuracy and sensitivity for early diagnosis of HCC by using combined serum GPC3 and alpha fetoprotein (AFP) were better than AFP alone. AFP is a glycoprotein, which is mainly synthesized by fetal liver cells and yolk sac. Elevated AFP in adulthood is considered as a pathological condition. AFP Levels are elevated in many diseases including HCC, stomach cancer, yolk sac tumors and so on. Besides, more than 70% liver cancer patients show positive AFP. Therefore, serum AFP is considered as the standard biomarker for clinical diagnosis of liver cancer. A study showed that AFP stimulated the progression of HCC by inhibiting human antigen R (HuR)-mediated Fas/FADD (Fas-associating protein with a novel death domain) apoptotic pathway (Chen et al., 2020). The diagnosis by AFP or GPC3 could be improved by combining GPC3 and AFP (El-Saadany et al., 2018). Another research showed that patients with high preoperative plasma level of GPC3 was more likely to undergo postoperative recurrence (Ofuji et al., 2017). Furthermore, it was shown that GPC3 promotes HCC growth by upregulating the expression of Wnt as well as insulin-like growth factors (Cheng et al., 2008). GPC3 was found to be significantly related to HCC tumorigenesis through Wnt-5,6,7, Yap8, transforming growth factor-β29 (TGF-β29), and human growth factor 10 (HGF10) signaling (Ho and Kim, 2011). Most importantly, apoptosis was induced in HCC cell lines when GPC3 was silenced, which suggest that GPC3 play an important role in HCC carcinogenesis (Liu et al., 2012).

The high expression of GPC3 preferentially in HCC suggest that it can be used as a target for immunotherapy and the GPC3 expression on the surface of HCC cells makes it a good target for antibody therapy (Wang Y. et al., 2018). GPC3-binding antibodies have indeed been developed for immunotherapies of HCC, such as unmodified antibodies, immunotoxin bound antibodies, GPC3/NK BsAb, BiTE, and other BsAb (Lipovšek et al., 2018). GPC3-S-Fab, is an antibody Fab fragment based BiAb, and recruits NK cells to eliminate GPC3 positive cancer cells by linking the Fab of anti-GPC3 antibody to anti-CD16 single domain antibody (Table 1 and Figure 1). In another BiAb, the GPC3/CD3 BiTE was also developed to recruit cytotoxic T lymphocyte (CTL) cells for clearance of GPC3^+^ HCC cells (Lampen et al., 2018; Table 1 and Figure 1). The therapeutic effect of BiTE depends on the concentration of GPC3/CD3 BiTE and the expression level of GPC3 on target cells. It has been demonstrated that the effect of BiTE is strongly GPC3-dependent *in vitro* and *in vivo* (Bi et al., 2017). In addition, GPC3-targeted CAR-T cells with CD28 co-stimulatory domain were also developed and showed anti-HCC effect in xenograft tumors (Guo et al., 2018). Furthermore, GPC3 peptide vaccine was also demonstrated to reduce recurrence rate of HCC patients (Sawada et al., 2016).

**TABLE 1:** Features of the tumor associated antigens of HCC.

	GPC3	TLS11a target	EpCAM	PD-1
Expression in normal tissues	Low	None	Low	T cells
Expression in HCC tumors	High in about 70%	High	High	PD-L1/2 high
Function	Cancer cell proliferation, migration, metastasis	TLS11a-BiTE Inhibits HCC cells Hep3B and Huh-7	A CSC marker	Inhibiting T cells
McAb scFv or gRNA sequence	Not found	5-ACA GCATCC CCA TGT GAA CAATCGCATTGTGATTGTTA CGGTTTCCGCCTCATGGACG TGCTGTTTTTTTTTT-SH-3	Not found	PD-1-gRNA-1: GTCTGGGCGG TGCTACAACT; PD-1-RNA-2: GGCCAGGATG GTTCTTAGGT.
Ref	Wang Y. et al., 2018	Hu Z. et al., 2018	Zhang et al., 2014	Guo et al., 2018

### TLS11a Aptamer

Aptamers are ssDNA or ssRNA that are selected via library screening using SELEX (systematic evolution of ligands by exponential enrichment). They can combine with various targets such as small dyes, proteins, peptides, the whole cells, tissues and toxins, as it can fold into a variety of forms of three-dimensional (3D) structure (Kumar Kulabhusan et al., 2020). An aptamer bispecific antibody TLS11a/CD3 (Sulfo-SMCC, Thermo Scientific Co.) has been constructed by combining TLS11a-SH or S2.2-SH with the anti-CD3-NH2 at 4°C for 24 h, and the non-crosslinked aptamers of the reaction mixture was removed by centrifuging at 14000 rpm for 10 min. TLS11a/CD3 can bind to both HCC cells and T cells by aptamer TLS11a and anti-CD3 single chain variable region, respectively (Figure 1). TLS11a/CD3 was capable to guide T cells to target and kill HCC cells with high specificity and affinity and was shown to repress the proliferation of HCC H22 cells *in vitro* and prolong mouse survival time by inhibiting the progression of xenograft tumor *in vivo* (Table 2). T cell proliferation and the production of multiple cytokines, such as interleukin-2 (IL-2), IL-6, IL-10, tumor necrosis factor-alpha (TNF-α), and interferon-gamma (IFN-γ), were significantly higher in the T cells + H22 + TL11a/CD3 + group than in the control group, which also supported the anti-tumor effect of TL11a/CD3. Compared to other lower concentration groups, TLS11a/CD3 (20 μg) group had the best effect in tumor inhibition and prolonging survival on the hepatoma xenograft model. The tumor inhibition efficacy of TLS11a/CD3 was found to be dose dependent (Hu Z. et al., 2018).

**TABLE 2:** BiAb-based immunotherapy against the tumor associated antigens of HCC.

Target	Drug	Methods	Effect	Adverse effect	Ref
GPC3 + HCC	GPC3/CD16 GPC3- S-Fab BiAb/BiTE	Cell culture, xenograft	Effective against tumors.	Not found	Wang Y. et al., 2018
H22 or BNL CL2 HCC	TLS11a aptamer/CD3 BiTE	Mouse xenograft	Mediated effective tumor lysis.	Poor stability, immunogenicity and high cost production	Hu Z. et al., 2018
EpCAM + HCC	anti-EpCAM BiTE (1H8/CD3)	Hep3B, Huh-7, mouse xenograft	Significantly suppressed tumor growth and CSC marker expression.	Gal-1 may be conducive to resistance to 1H8/CD3-induced lysis.	Zhang et al., 2014

### EpCAM-BiTE

Epithelial cell adhesion molecule is a transmembrane glycoprotein and its expression was increased in HCC tissues compared with adjacent normal liver tissues (Schmelzer et al., 2006). EpCAM expression in HCC was positively correlated with chemotherapy resistance and recurrence (Li Y. et al., 2016). Importantly, EpCAM-positive cells showed characteristics of CSC and EpCAM^+^AFP^+^ HCC was associated with poor prognosis. High tumorigenicity, high colony formation and low differentiation potency were found in EpCAM-positive HCC cell line PLC/PRF/5 and the proliferation and invasiveness of HCC cells were significantly reduced when EpCAM expression was downregulated (Kimura et al., 2014). EpCAM was found to be one of the Wnt-β-catenin signaling direct transcription target in normal human hepatocytes and hepatoma cells (Yamashita et al., 2007). CSC enrichment promotes the HCC tumorigenesis by Wnt-β-catenin signaling (Pandit et al., 2018). In response to Wnt-β-catenin signaling antagonists (natural compounds PKF118-310, PKF115-584, and CGP049090) (Lepourcelet et al., 2004), EpCAM expression was significantly decreased, suggesting Wnt-β-catenin signaling promoted EpCAM expression (Yamashita et al., 2007). A Study found that liver cancer CSCs with EpCAM-high upregulated the expression of carcinoembryonic antigen-related adhesion molecule 1 (CEACAM1) to resist the natural killer (NK) cell mediated cytotoxicity (Park et al., 2020). Moreover, EpCAM positive circulatory stem-like cells were associated with unfavorable prognosis of HCC patients who underwent radical resection. Therefore, EpCAM is considered as a CSC marker and a potential target for immunotherapy of HCC (Yamashita et al., 2007, 2009).

An anti-EpCAM BiTE, 1H8/CD3, has been constructed and was shown to inhibit the growth of xenograft tumors from HCC cell lines Hep3B and Huh-7 *in vivo* (Figure 1). Xenografts from the 1H8/CD3 treated mice showed decreased expression of majority CSC markers. However, the function of 1H8/CD3 was inhibited when galectin-1 (Gal-1) was overexpressed in HCC tumors (Zhang et al., 2014).

### PD-1 Immune Check Point Inhibitor

Programmed death-1 is a transmembrane receptor that belongs to the immunoglobulin super family (IgSF) (Ohaegbulam et al., 2015) and is mainly expressed on the surface of CD3^+^ T lymphocytes and NK cells, and functions as an inhibitory receptor (Gros et al., 2014). A study showed that both T-cell immunoglobulin domain and mucin domain containing molecule-3 (TIM-3) and PD-1 were highly expressed on the infiltrating lymphocytes of hepatitis B-related HCC tumors and adjacent tissues (Li Z. et al., 2016). PD-L1/2 on the surface of cancer cells inhibits activation and proliferation of T cell, resulting in cancer cell escape from the immune response (Zou et al., 2016). Besides, a study showed that MYC (one of the frequently altered ongenes in patients with HCC) in the tumor suppressor p53−/− HCC upregulated β-catenin signaling to promote immune escape. More importantly, the activation of β-catenin repressed the recruitment of DCs, accelerated the immune escape and caused the resistance of the anti-PD-1 therapy (Ruiz De Galarreta et al., 2019). In addition, the up-regulation of Toll-like receptor 9 (TLR9) by PD-L1 induces immune escape in HCC (Zhou et al., 2020). Researchers demonstrated that blocking PD-1 or PD-L1 induce activation of T cells with increased IFN-γ release and T cell proliferation (Figure 1). PD-1 inhibits the consumption of oxygen, represses glutaminolysis and glycolysis of the activated T cells and reshapes their metabolism appropriately to change T cell differentiation. Besides, the PD-1 pathway induces PD-L1 by proinflammatory signal to inhibit the effector T cells and maintain self-tolerance. In addition, low levels of PD-1 expression are needed to suppress the expansion of T-cell as well as the IFN-γ, IL-2, and TNF-α production (Boussiotis, 2016). High PD-L1 expression in HCC tumors were an index of unfavorable prognosis for HCC patients who underwent surgical resection (Wang et al., 2017) and the level of serum PD-L1 is also positively correlated with HCC stages and mortality risk (Finkelmeier et al., 2016).

PD-1^+^ tumor infiltrating lymphocytes were an effective prognostic biomarker to predict the survival benefit for HCC patients who underwent the immunotherapy with cytokine induced killer (CIK) (Chang et al., 2018). PD-1 knockout was found to significantly enhance the anti-HCC efficacy of CIK cells. Study have shown that the combination of human telomerase reverse transcriptase (hTERT) transduction and PD-1 knockout of CIK cells improved the anti-HCC efficacy of CIK cells (Huang et al., 2018). In addition, PD-1 disruption was found to enhance the anti-HCC effect of GPC3 CAR-T cells in NOD-scid-IL-2Rγ/−/− (NSG) mice *in vivo* (Guo et al., 2018). Furthermore, Nivolumab, a complete human IgG4 monoclonal antibody against PD-1, has been approved by the United States Food and Drug Administration (FDA) for late stage melanoma and metastatic non-small cell lung cancer. Research have showed that Nivolumab also get an acceptable effectiveness in HCC patients and may become an alternative therapy for HCC patients who have failed routine treatments (Feng et al., 2017). Besides, pembrolizumab, another FDA approved anti-PD-1 antibody, also demonstrated that the anti-tumor activity and safety in previously treated patients with HCC in clinical trials (Finn et al., 2020a).

### VEGF

The vascular endothelial growth factor (VEGF), a signaling ligand released by epithelial cells, is an important positive angiogenesis regulator (Yang et al., 2014; Zhang et al., 2020) and VEGF signaling plays a significant role in inhibiting the apoptosis and promoting proliferation in tumor cells (Liu et al., 2016). Highly vascularized tissue of adult liver is an important feature for its function, each liver cell is lined with sinusoidal endothelial cells (SEC) on both sides. It was reported that SEC fenestration was regulated by hepatocyte-mediated VEGF signaling during liver angiogenesis (Carpenter et al., 2005; Sharma et al., 2020). In addition, a study demonstrated that HCC shared the immune microenvironment and stromal microenvironment with fetal liver and suggested that VEGF and NOTCH signaling play important role in the maintenance of onco-fetal ecosystem. Importantly, it was shown that VEGF was also related with the metastasis and recurrence of HCC (Minata et al., 2013). Moreover, the down-regulation of exosmosis tumor C-Type Lectin Domain Family 3 Member B (CLEC3B) could accelerate the angiogenesis and metastasis of HCC by VEGF and AMP-activated protein kinase (AMPK) pathways (Dai et al., 2019). Research has indicated that tumor immune microenvironment and VEGF signaling pathway in HCC patients are synergistically activated, which suggests a prominent mechanism of combined therapy including immune checkpoint blockades (ICBs) and anti-VEGF drugs (Liu et al., 2020). Atezolizumab and bevacizumab are PD-L1 inhibitor and VEGF inhibitor, respectively, and a recent study of IMBrave150 trial indicated that comparing with sorafenib, atezolizumab in combination with bevacizumab improved overall response rate, overall survival and progression-free survival dramatically in patients with unresectable HCC (Finn et al., 2020b; Hack et al., 2020). Furthermore, FDA approved the combination of atezolizumab with bevacizumab as a new first-line treatment for advanced or unresectable HCC patients (Liu et al., 2020). The IMBrave150 trial also suggested that double blockade of PD-L1/VEGF can effectively reduce the recurrence of HCC by creating a more immunologically advantageous microenvironment (Hack et al., 2020). Apatinib is a specific inhibitor of VEGF-receptor 2 (VEGFR2) (Tian et al., 2011) and a recent study showed that apatinib blocked the VEGF and PI3K/AKT signaling pathways to inhibit the migration, invasion and angiogenesis of HCC cells (Song et al., 2021). Besides, lots of clinical trials involving as the VEGFR2 inhibitor apatinib in HCC are ongoing as showed in Table 3, most of which are exploring the combination of apatinib with other drugs or therapies to treat HCC to extend the survival time or to reduce the adverse side-effects. Furthermore, ramucirumab, another inhibitor of VEGF-receptor 2, was showed to have survival benefit in an age subgroup with safety tolerance, which supported its use in late stage HCC with elevated AFP, regardless of age (Kudo et al., 2020). Therefore, as a second line drug, ramucirumab was approved by FDA for the advanced HCC patients with AFP ≥ 400 ng/mL after sorafenib treatment (De Luca et al., 2020).

**TABLE 3:** Ongoing clinical trials involving Apatinib (VEGFR2 inhibitor) in HCC.

Name	Trail ID	Phase	Study population	Intervention	Status
Apatinib	NCT03046979	II	Advanced HCC patients	Apatinib	Unknown
Apatinib and TACE	NCT03066557	Not Applicable	HCC patients	TACE and Apatinib	Unknown
Apatinib	NCT01192971	II	Advanced HCC patients	Apatinib	Completed
Apatinib	NCT02727309	I/II	Advanced HCC patients	Apatinib after TACE	Unknown
Apatinib and Camrelizumab	NCT04521153	Not Applicable	Resectable HCC patients	Camrelizumab and Apatinib Mesylate Procedure: TACE treatment and radical surgery	Recruiting
SHR-1210 and Apatinib	NCT04297202	II	HCC patients	Apatinib combined with SHR-1210 injection	Recruiting
Apatinib and Capecitabine	NCT03114085	II	Advanced HCC patients	Capecitabine and Apatinib compared with Apatinib	Unknown
Cryoablation, Camrelizumab and Apatinib	NCT04724226	II	Advanced HCC patients	Cryoablation, Camrelizumab, Apatinib	Not yet recruiting
SHR-1210 Plus Apatinib	NCT04014101	II	Advanced stage HCC	SHR-1210 and Apatinib	Recruiting
Apatinib	NCT02772029	I/II	Advanced HCC Patients After First-line Treatment Failure	Apatinib Mesylate Tablets	Unknown
SHR-1210 Plus Apatinib	NCT03722875	Not Applicable	BCLC B and C stage HCC after surgery	SHR-1210 and Apatinib	Unknown
Sintilimab, Apatinib and Capecitabine	NCT04411706	II	Advanced HCC patients	Sintilimab Combined With Apatinib and Capecitabine	Recruiting
Apatinib	NCT03511703	II	Advanced HCC	Postoperative adjuvant Apatinib vs. TACE, chemotherapy drugs + iodized	Unknown
Apatinib and Camrelizumab	NCT04191889	II	C-staged HCC patients	Hepatic Arterial Infusion with Apatinib and Camrelizumab	Recruiting
Apatinib plus radiotherapy	NCT03520257	II	HCC patients with BCLC-C stage I and II portal vein tumor thrombus	Apatinib plus radiotherapy vs. Apatinib	Unknown
Camrelizumab, Apatinib and Oxaliplatin	NCT04850040	II	Patients with potentially resectable HCC	Apatinib Mesylate, Camrelizumab and Oxaliplatin	Not yet recruiting
Camrelizumab and Apatinib	NCT04701060	II	Resectable primary HCC patients	Camrelizumab Combined With Apatinib	Recruiting
Camrelizumab, Apatinib and chemotherapy	NCT04479527	II	Advanced HCC patients	(cTACE or DEB-TACE + FOLFOX regimen HAIC) combined with Camrelizumab and Apatinib	Not yet recruiting
Apatinib and SHR-1210	NCT02942329	I/II	HCC or gastric cancer patients	Apatinib and SHR-1210	Unknown
Camrelizumab and Apatinib	NCT04826406	II	HCC patients previously treated with immune checkpoint inhibitors	Camrelizumab combined with Apatinib regimen	Recruiting
SHR-1210 and Apatinib	NCT03793725	II	Patients with unresectable HCC	SHR1210 combined with Apatinib	Unknown
Apatinib and SHR-1210	NCT03839550	II	HCC patients with high risk of recurrence after radical resection	Hepatic Arterial Infusion (HAI) of Apatinib Mesylate + PD-1 antibody SHR-1210.	Not yet recruiting
Camrelizumab Plus Apatinib	NCT04639180	III	Patients with HCC at high risk of recurrence after surgical	Camrelizumab plus Apatinib as adjuvant therapy in HCC	Not yet recruiting
M1-c6v1, SHR-1210 and Apatinib	NCT04665362	I	HCC patients	Recombinant oncolytic virus M1-c6v1, anti-PD-1 antibody SHR-1210, and Apatinib	Not yet recruiting
Camrelizumab plus Apatinib	NCT04035876	I/II	Downstaging/bridging of HCC patients before liver transplant	Camrelizumab (SHR-1210) and Apatinib combination	Recruiting
Radiotherapy, and Apatinib	NCT03732105	II	HCC patients received curative resection with microvascular invasion	Radiotherapy, Apatinib, or radiotherapy + Apatinib	Not yet recruiting
TACE, Camrelizumab and Apatinib	NCT04559607	Not Applicable	Intermediate and advanced HCC patients	TACE combined with Camrelizumab and Apatinib	Recruiting
SHR-1210 and Apatinib	NCT03463876	II	Advanced HCC patients	SHR 1210 + Apatinib	Active, not recruiting
Camrelizumab and Apatinib	NCT04523662	II	Advanced liver cancer patients	Carrelizumab combined with Apatinib Mesylate and radiotherapy	Not yet recruiting
Apatinib	NCT02329860	III	HCC patients after systemic therapy	Apatinib or placebo	Completed
Apatinib and TACE	NCT02702323	II/III	Patients with pulmonary metastasis of liver cancer	Apatinib combined with TACE	Unknown
Thermal Ablation, Apatinib and Carilimub	NCT04204577	II	Advanced liver cancer patients	Thermal ablation combined with Apatinib and Carilimub	Recruiting
Apatinib	NCT03261791	II	HCC patients with PVTT who underwent radical resection	Adjuvant therapy with Apatinib	Unknown
SHR-1210 and Apatinib	NCT03764293	III	Advanced HCC patients	SHR-1210 in Combination With Apatinib as first-line therapy vs. Sorafenib	Recruiting

## Clinical Trials of HCC Immunotherapy

A series of anti-GPC3 chimeric antigen receptor modified T cells (GPC3-CAR-T) had being tested in phase I/II trials (Table 4). In addition, a humanized anti-human GPC3 antibody, GC33, was tested in clinical trials to examine the pharmacokinetics, dosage and duration of treatment, safety and tolerability, and antitumor activity in GPC3 high expression hepatoma cells (Tables 4, 5). The adverse reactions of GC33 were mainly fatigue (50%), constipation (35%), headache (35%), and hyponatremia (35%), most of which were grade 1 or 2. The number of peripheric NK cells was decreased after GC33 treatment, but no increased incidence of infection was observed. These research have showed that GC33 had potential antitumor activity in patients with high GPC3 expression HCC tumors and provided a preliminary clinical basis for further trials in advanced HCC (Zhu et al., 2013). However, grade 3 adverse events were shown as blood pressure increase, lymphocyte and platelet count decrease in two or more patients when the same agent was tested in Japan (Ikeda et al., 2014). Furthermore, a phase I clinical trial was performed on a GPC3 derived peptide vaccine (Table 5; Sawada et al., 2012). The peptide vaccine caused grade III hematologic adverse events (impaired liver function) in 4 out of 33 patients, although lymph node regression in 24/33 patients and liver tumor disappearance in 2/33 patients were observed (Sawada et al., 2012).

**TABLE 4:** Ongoing clinical trials involving GPC3 in HCC.

Name	Trail ID	Phase	Study population	Intervention	Status
GC33	japicCTI 101255	I	HCC with no preferred treatment	GC33	Unknown
GPC3 CAR-T	NCT02395250	I	GPC3 + HCC patients	GPC3 CAR-T cells	Completed
GPC3 CAR-T	NCT02723942	I/II	GPC3 + HCC patients	GPC3 CAR-T cells	Completed
GPC3 CAR-T	NCT03084380	I/II	GPC3 + HCC patients	Anti-GPC3 CAR-T cells	Unknown
GLY CAR-T	NCT02905188	I	Unresectable, recurrent metastatic GPC3 + HCC patients	GLYCAR T cells; Fludarabine	Recruiting
GPC3 CAR-T	NCT03884751	I	Late stage HCC patients, unresectable	GPC3 CAR-T cells	Recruiting
GPC3-T2 CAR-T	NCT03198546	I	GPC3 + advanced HCC patients	GPC3 and/or TGFβ targeting CAR-T cells	Recruiting
GPC3 CAR-T	NCT04506983	I	GPC3 + HCC patients after failure or intolerance of first-line treatment	GPC3 CAR-T cells	Not yet recruiting
GPC3 CAR-T	NCT03146234	Not Applicable	GPC3 + relapsed or refractory HCC patients	GPC3 CAR-T cells	Completed
GPC3 CAR-T	NCT04121273	I	GPC3 + advanced HCC patients	GPC3 CAR-T cells	Recruiting
GPC3 CAR-T	NCT03980288	I	Refractory or intolerant to current standard systemic treatment, GPC3 + advanced HCC patients	GPC3 CAR-T cells	Recruiting
GPC3 CAR-T	NCT02715362	I/II	Unresectable, at least one prior standard of care chemotherapy, GPC3 + advanced HCC patients	TAI-GPC3 CART cells	Unknown
GPC3 CAR-T	NCT03130712	I/II	GPC3 + advanced HCC patients with one prior standard of chemotherapy or surgery	GPC3 CART cells	Unknown
CT0180 Cells	NCT04756648	I	GPC3 + advanced HCC patients	CT0180 humanized anti GPC3 autogenous T cell injection	Not yet recruiting
GPC3 CAR-T	NCT04121273	I	GPC3 + advanced HCC patients	CAR-T cell immunotherapy	Recruiting
ECT204 T-Cell therapy (ARYA3)	NCT04864054	I/II	GPC3 + adults advanced HCC patients with failure or intolerance of at least two different anti-HCC systemic agents	ECT204 T cells	Not yet recruiting
GC33 (RO5137382)	NCT01507168	II	Unresectable advanced or metastatic GPC3 + HCC patients	GC33	Completed
GPC3 CAR-T	NCT02959151	I/II	GPC3 + advanced liver malignancy	CAR-T cell	Unknown

**TABLE 5:** Published clinical trials involving GPC3, ALK-1, and PD-1 in HCC immunotherapy.

Drug	Combination	Route, dose	Enrollment	Efficacy	Adverse effect	Phase	Ref
GPC3 derived peptide vaccine	None	Intracutaneously, on days 1, 15 and 29, at doses 0.3, 1.0, 3.0, 10, 30 mg/body surface area.	Non-randomized, open label	24/33 lymph node regression, 2 liver tumors disappeared.	Grade III hematologic adverse events (impaired liver function) in 4 patients	I	Sawada et al., 2012
GC33	75% patients received sorafenib	Dose escalation, 2.5–20 mg/kg, weekly i.v.	Multicenter, open label, single arm	AFP levels decreased or stabilized	Grade III, NK cell numbers in plasma decreased.	I	Zhu et al., 2013
Anti-ALK-1 McAb PF-03446962	Antiangiogenic or sorafenib therapy	1 h iv on days 1 and 29 and every 2 weeks thereafter, RP2D of 7 mg/kg.	Single-arm	Disease control rate at 12 weeks was 29%.	Grade III Thrombocytopenia in 33%, grade IV abdominal pain in 1 patient.	I	Simonelli et al., 2016
SHR-1210, an anti-PD-1 McAb	Apatinib, a VEGFR2 Inhibitor	Oral apatinib once-daily combined with SHR-1210 administered intravenously every 2 weeks.	Single center, open label.	Objective response rate is 30.8%, partial response is 50%.	Grade III Lipases rise (6.7%), preumonitis (20%) Hypertension (15.2%), increased AAT 15.2%.	Ia and Ib	Xu et al., 2019

PF-03446962 is an activin receptor-like kinase-1 (ALK-1) monoclonal antibody. A phase I clinical trial had explored the safety, pharmacokinetics and antitumor activity of PF-03446962 in total of 24 HCC patients. The most common treatment-related adverse events were thrombocytopenia (33.3%), fatigue (29.2%), shivering (16.7%), fever epistaxis and anemia, and ENT-associated telangiectasia (2 patients). Based on the trial, the disease control rate was 29% (Simonelli et al., 2016) and a phase II trial was suggested to have a dose of 7 mg/kg of PF-03446962 for single drug treatment of HCC patients.

Antibodies against PD-1 and its ligand PD-L1 have shown anti-tumor effects in many cancers including liver cancer (Apolo et al., 2017). SHR-1210 is an anti-PD-1 antibody and phase Ia and Ib clinical trials that combined SHR-1210 with apatinib (VEGFR2 inhibitor) for the treatment of advanced HCC, stomach and esophageal cancers were performed. Phase Ia was designed to identify the maximum tolerated dose (MTD) and the recommended phase II dose (RP2D) of SHR-1210 when combined with apatinib. The combination of SHR-1210 and apatinib showed controllable toxicity in HCC and GC/EGJC patients with recommended single dose. The RP2D of SHR-1210 in the apatinib combined treatment was 250 mg, demonstrating encouraging clinical activity in patients with advanced liver cancer. SHR-1210 combined with apatinib showed the objective response rate of 30.8% (95% CI: 17.0–47.6%) as observed in 39 patients. Of the 16 evaluable HCC patients, 8 patients obtained partial responses (50.0%, 95% CI: 24.7–75.4%). The grade 3 adverse events observed are hypertension (15.2%) and elevated aspartate aminotransferase (AAT, 15.2%) (Xu et al., 2019). In brief, the combination of SHR-1210 and apatinib demonstrated encouraging results in patients with advanced liver cancer. Moreover, there are lots of ongoing clinical trials involving PD-1 treatment in HCC as shown in Supplementary Table 1, which mostly explore the efficacy and safety of new drugs and new therapies of existing drugs on HCC. However, most of these clinical trials are in phase I/II without published results.

## Summary and Future Perspectives

Hepatocellular carcinoma is one of the most common and lethal tumors worldwide. Small molecule targeted drugs, such as sorafenib, have only about 3 months survival benefit due to drug resistance to cancer stem cells (Xin H. W. et al., 2013). Therefore, further improvement of HCC therapy is urgent. Tumor immunotherapy has shown significant potential. GPC3 based HCC immunotherapies included GPC3/NK BsAb, GPC3/BiTE, GPC3/CAR-T, GPC3 mAb, and GPC3 peptide vaccine. These HCC immunotherapies have showed promising results in preclinical studies, ongoing phase I/II clinical trials and published phase I/II clinical trials. The published clinical trials demonstrated their preliminary safety and effectiveness, which warranted for their phase II trials in future. In addition, TLS11a aptamer-BiTE and EpCAM/BiTE showed their effectiveness in eliminating HCC tumors in mouse models. Anti-PD-1 antibody was also reported to suppress HCC progression in mouse xenograft and to be preliminarily safe and effective in clinical trials.

Nevertheless, most of the current specific antibodies applied in the HCC treatment are in preclinical experimental stage or early phase clinical trials. The antibodies that worked on animals may not work well on humans due to heterogeneity and complex immunogenicity, as different species have different immune rejection response and human body may produce rejection reaction to the animal source antibodies in different degree. Their effectiveness and safety need to be improved. The antibodies used in the immunotherapy had immunogenicity, poor stability, and high cost, which may limit their clinical application. CAR-T modification, in combination with the disruption of inhibitory immune checkpoints, represents a promising method of tumor immunotherapy. Moreover, the HCC immunotherapy has not achieved significant results in clinical trials and further effective approaches are needed to explore for HCC immunotherapy. In future, we suggest that dendritic cell (DC) based tumor immunotherapy may be studied in HCC, as DC-derived exons was found to be involved in antigen presentation during anti-tumor immune response, besides, DC is the most important monitoring sentinel cell in tumor microenvironment (Wang et al., 2020). Oncolytic viruses may also be applied for HCC tumor immunotherapy. It is superior to conventional tumor treatments due to a relatively shorter period of treatment, reduction of toxicity, as well as the possibility of targeting micro-metastases (Wu et al., 2018; Cai et al., 2020; Wang et al., 2020). Engineered viral envelope glycoproteins can specifically target tumors (Liu X. Q. et al., 2018) and the technology of clustered regularly interspaced short palindromic repeats/CRISPR-associated protein 9 (CRISPR/Cas9) genomic editing has prominently promoted the study of oncolytic viruses (Wang D. et al., 2018).

## Author Contributions

S-LH, L-MY, and H-WX designed this research. S-LH contributed to literature search and drafted the manuscript. S-LH, Y-MW, Q-YW, G-GF, F-QW, L-MY, X-HZ, and H-WX revised and edited the manuscript. L-MY, X-HZ, and H-WX approved the final version of the manuscript. All authors contributed to the article and approved the submitted version.

## Conflict of Interest

The authors declare that the research was conducted in the absence of any commercial or financial relationships that could be construed as a potential conflict of interest.
